# Mélanome des tissus mous: cas clinique

**DOI:** 10.11604/pamj.2017.28.107.10316

**Published:** 2017-10-04

**Authors:** Rachid Frikh, Siham Oumakhir, Hafsa Chahdi, Mohammed Oukabli, Abderrahmane Albouzidi, Noureddine Baba, Naoufal Hjira, Mohammed Boui

**Affiliations:** 1 Service de Dermatologie, Hôpital Militaire d’Instruction Mohammed V, Rabat, Maroc; 2 Service d’Anatomie Pathologique, Hôpital Militaire d’Instruction Mohammed V, Rabat, Maroc; 3 Faculté de Médecine et de Pharmacie, Université Mohamed V, Rabat, Maroc

**Keywords:** Mélanome des tissus mous, tumeur pédiculée, extrémités, Soft tissue melanoma, pedicled tumor, extremities

## Abstract

Le mélanome des tissus mous a été décrit en 1965 par Enzinger sous le nom de sarcome à cellules claires. En 1983, Chung et Enzinger le rebaptisent mélanome des parties molles en raison de similitudes immunohistochimiques avec le mélanome. Nous rapportons un cas de cette forme rare de mélanome, chez un jeune homme de 22 ans qui avait une lésion molluscoïde de la cheville sans signes cliniques de malignité, et dont l’examen histologique a permis de retenir ce diagnostic.

## Abstract (en)

Soft tissue melanoma was first described by Enzinger in 1965 under the name of clear cell sarcoma. In 1983, Chung and Enzinger renamed it soft tissue melanoma due to its immunohistochemical similarities with melanoma. We here report the case of a 22-year old young man with this rare type of melanoma, presenting with molluscoid lesion on his ankle without any clinical sign of malignancy. Histology examination confirmed the diagnosis of soft tissue melanoma.

## Introduction

Le mélanome des tissus mous (MTM) a été décrit en 1965 par Enzinger sous le nom de sarcome à cellules claires à partir d’une série de 21 malades, le plus souvent jeunes, qui avaient une tumeur des extrémités, étroitement liée aux tendons ou aux aponévroses. Nous rapportons l’observation d’un jeune patient qui avait une lésion asymptomatique de la cheville, apparemment bénigne, mais dont l’histologie s’est montrée très atypique.

## Patient et observation

**Figure 1:**
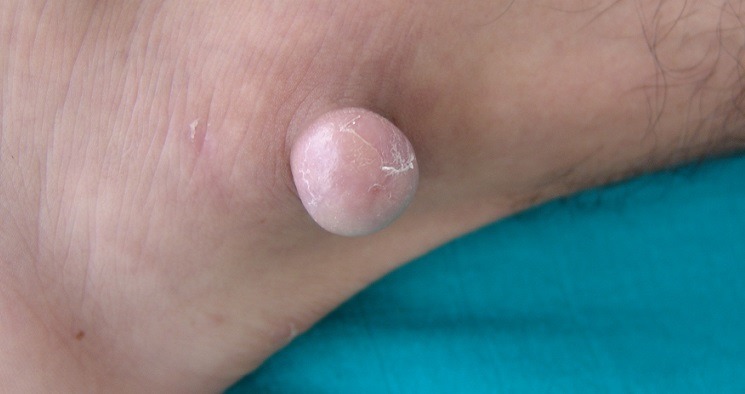
Lésion molluscoïde, couleur peau normale, en regard de la malléole externe gauche, d’apparence bénigne

**Figure 2:**
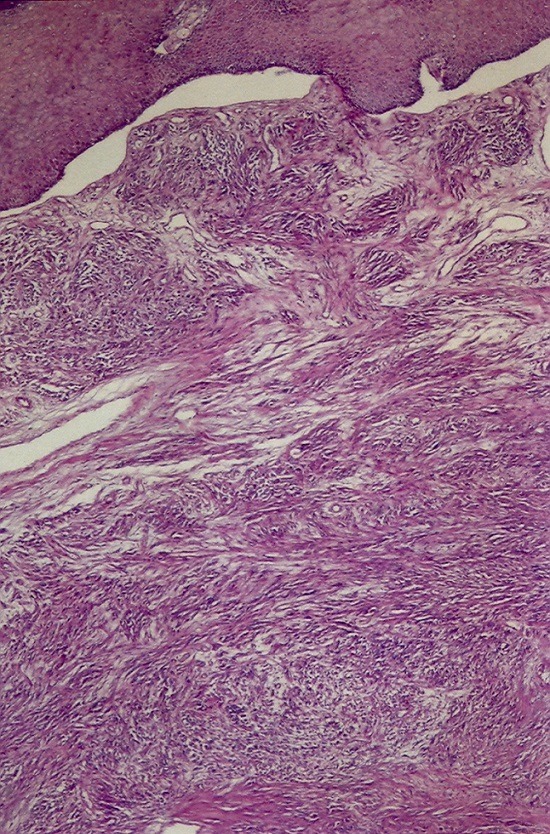
Aspect histologique de la pièce d’exérèse au faible grossissement (HEx40)

**Figure 3:**
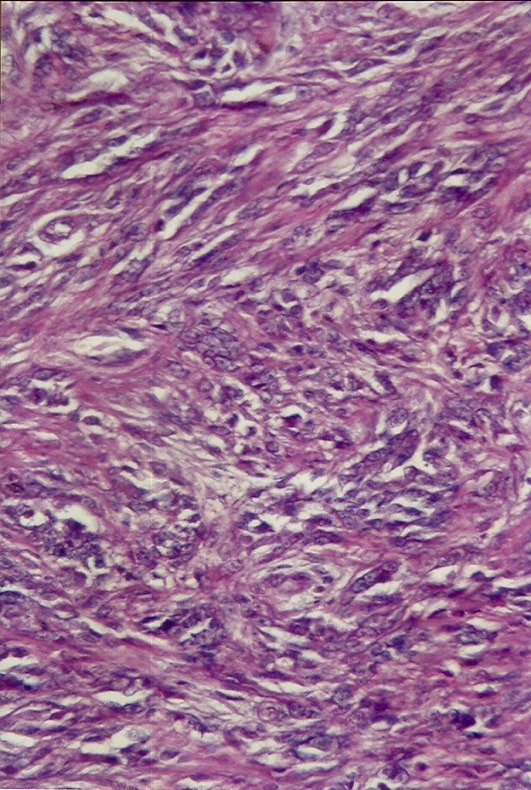
Aspect histologique de la pièce d’exérèse au fort grossissement (HEx200)

Mr H. âgé de 22 ans, sans antécédents pathologiques, avait consulté en Novembre 2006 pour une lésion exophytique asymptomatique de la malléole externe gauche, évoluant depuis 3 ans, augmentant très progressivement de volume, sans gêne fonctionnelle. L’examen initial a objectivé une lésion molluscoïde de 3 cm de diamètre, couleur peau normale, pédiculée, à surface lisse et indolore (Figure 1). L’exérèse chirurgicale était totale avec étude histologique de la pièce (Figure 2, Figure 3). L’évolution a été marquée par une ulcération torpide sur le site opératoire, sans tendance à la cicatrisation spontanée. A l’étude microscopique, on a observé un tissu cutané et sous-cutané infiltrés par une prolifération tumorale constituée de cellules essentiellement fusiformes, présentant des noyaux fortement nucléolés, relativement réguliers par ailleurs. Il n’a pas été observé de nécrose tumorale, mais plutôt quelques figures mitotiques. Les immunomarquages réalisés montrent une positivité diffuse de la protéine S100 et de la vimentine, une positivité plus focale de l’HMB45 et pour le MélanA. L’index de prolifération évalué par Mib1, est d’environ 10 à 20% des cellules tumorales.

L’étude histologique a attesté que l’exérèse était complète avec passage en zone saine, et que les limites de la tumeur étaient régulières. Le diagnostic de **mélanome des tissus mous** a été retenu. La recherche du transcrit spécifique de fusion, réalisée à l’institut Bergonié, était non concluante, vu que les prélèvements ont été fixés dans le liquide de Bouin. Le bilan d’extension a objectivé la présence de deux micronodules pulmonaires des segments VI droit et gauche, sans métastases ganglionnaires ni cliniques ni échographiques. Sur le plan thérapeutique, une exérèse élargie sur l’ulcération résiduelle a été réalisée en vue d’élimination des résidus tumoraux. Après étude histologique des recoupes de la deuxième pièce, une greffe de peau mince a été préconisée pour couvrir la perte de substance engendrée. L’abstention thérapeutique avec surveillance régulière des nodules pulmonaire a été décidée, avec un aspect toujours stationnaire, des nodules pulmonaires, après deux ans de recul.

## Discussion

Le mélanome des tissus mous (MTM) a été décrit en 1965 par Enzinger sous le nom de sarcome à cellules claires à partir d’une série de 21 malades, le plus souvent jeunes, qui avaient une tumeur des extrémités, étroitement liée aux tendons ou aux aponévroses [1]. La plus grande série de la littérature, publiée par Chung et Enzinger en 1983, a colligé 141 cas provenant des dossiers de l'Institut d’Anatomo-pathologie des Forces Armées Américaines [2]. Ces auteurs ont proposé la dénomination de mélanome des tissus mous, vu les similitudes histologiques avec le mélanome. La tumeur touche presque également l’homme et la femme, avec un sex-ratio F/H de 1,13 dans la série de Chung et Enzinger [2]. De siège ubiquitaire, le MTM siège principalement sur les membres inférieurs (69 p. 100 des cas), et surtout les pieds. L’atteinte des membres supérieurs est moins fréquente avec, là aussi, une nette prédominance pour l’atteinte distale [2].

Dans la majorité des cas publiés dans la littérature, le patient consulte pour une masse ayant augmenté progressivement de volume, indolore ou discrètement sensible. Ceci explique le délai souvent long entre l’apparition de la tumeur et la première consultation [3-6]. La peau en surface est généralement inchangée, parfois tendue, et classiquement il n’y a pas d’invasion osseuse sous-jacente, confirmée par les radiographies osseuses, qui peuvent montrer rarement un aspect lytique [7]. Les calcifications intratumorales sont rares. La tumeur prend à l’imagerie par résonance magnétique, en pondération T1 un aspect homogène et isointense au muscle, alors qu’en T2 elle apparaît hypointense à centre proéminent, plus hétérogène et d’intensité différente à celle du muscle. Ces aspects ne permettent que d’évoquer le diagnostic, sans grande spécificité, vues les similitudes clinico-radiologiques avec les tumeurs des gaines et tendons [8].

Histologiquement, le MTM réalise une prolifération monomorphe de grosses cellules polygonales ou fusiformes, à cytoplasme pâle ou faiblement éosinophile, contenant un noyau vésiculé, au sein duquel, on observe un ou plusieurs nucléoles basophiles proéminents. Ces cellules prennent parfois un aspect épithélioïde. Elles sont groupées en amas, en faisceaux ou en lobules, délimités par de fins septa fibreux [9, 10]. Chung et Enzinger notent dans leurs descriptions initiales, la présence dans 72p. 100 des cas de mélanine intra cytoplasmique [2]. Les mitoses sont généralement peu nombreuses.

Notre observation réunit tous les éléments cliniques et histologiques du diagnostic. Les immunomarquages permettent d’individualiser cette entité, avec une positivité pour les marqueurs mélanocytaires (HMB45, protéine S100, Melan A). Les marqueurs constamment négatifs sont la cytokératine, EMA, LCA et la desmine [10, 11]. Le profil immunohistochimique de notre patient était plus en faveur du MTM avec une expression de la vimentine, de l’HMB45 et la PS100, et absence d’expression de la cytokératine et de la desmine.

La confirmation diagnostique est apportée par l’étude cytogénétique, non concluante chez notre patient, qui objective une translocation réciproque t(12;22)(q13;12), révélée par RT-PCR (reverse transcriptase polymerase chain reaction) ou FISH (fluorescent in situ hybridization, qui semble être plus sensible: jusqu’à 95p.100 des cas) [12]. Cette translocation entraîne la formation d’un gène fusion EWSR1/ATF1, par fusion de la portion 3’ de l’oncogène du sarcome d’Ewing EWSR1, avec la portion 3’ de l’oncogène du facteur d’activation de la transcription ATF1 sur le chromosome 12. Deux transcrits protéiques chimériques issues de ce gène hybride ont été décrit, responsables de la prolifération et de la transformation cellulaire [13-16].

Le principal diagnostic différentiel est le sarcome épithéloïde des parties molles, qui se présente aussi comme une masse sous cutanée, souvent située sur l’extrémité d’un membre, en particulier la main [17]. La protéine S100 est parfois faiblement positive, mais surtout 1’HMB45 est toujours négatif. Après la réalisation des marqueurs immunohistochimiques, le diagnostic différentiel peut se poser avec une localisation métastatique d’un mélanome passé inaperçue, et c’est l’étude cytogénétique qui départage catégoriquement les deux entités, avec absence constante de la translocation t(12;22)(q13;12), et positivité de plus en plus décrite, d’une mutation du BRAF gène qui serait spécifiques des tumeurs de la lignée mélanocytaire, notamment le mélanome [18].

L’évolution de cette tumeur est sombre, se rapprochant de celle des sarcomes, avec un taux de récidives locales élevé (47 p. 100 [17]), contrairement au mélanome où ce taux est de 5 à 10p.100 selon l’épaisseur ou indice de Breslow. Les métastases sont par ordre de fréquence décroissant, ganglionnaire, pulmonaire, cutanée, osseuse et hépatique, et seraient plus fréquentes quand la tumeur dépasse 5 cm de diamètre. L’existence d’une métastase ganglionnaire loco-régionale prédispose au risque de développement de métastases à distance [10].

Les facteurs de mauvais pronostic sont, en plus de la taille supérieure à 5cm, la présence de nécrose, la survenue de métastases, ou de récidives après exérèse chirurgicale [10]. Cette dernière quand elle est complète et « large »est généralement suffisante. L’amputation du segment du membre concerné a été préconisée dans certains cas, sans pour autant que sa supériorité par rapport au traitement conservateur soit prouvée. La réalisation de l’étude du ganglion sentinelle semble justifiée et de plus en plus proposée pour détection précoce de métastases ganglionnaires occultes, et dont la prise en charge semblerait améliorer le pronostic [19, 20].

Les compléments thérapeutiques radio-chimiothérapiques préconisés par certains auteurs, sont difficiles à analyser, et avec des indications non encore bien établies et validées. Le cours évolutif de cette tumeur n’est pas uniquement inconnu, mais surtout difficile à prédire dans l’état actuel des connaissances.

## Conclusion

Le mélanome des tissus mous est une tumeur mélanocytaire rare, différente du mélanome par son terrain, ses caractéristiques cliniques et évolutives, de diagnostic difficile, avec un pronostic qui se rapproche à celui des sarcomes.

## Conflits d’intérêts

Les auteurs ne déclarent aucun conflit d’intérêts.
